# Antiulcer Effect of Honey in Nonsteroidal Anti-Inflammatory Drugs Induced Gastric Ulcer Model in Rats: A Systematic Review

**DOI:** 10.1155/2018/7515692

**Published:** 2018-07-15

**Authors:** Annuar Fazalda, Adam Quraisiah, Mohd Fahami Nur Azlina

**Affiliations:** Department of Pharmacology, Faculty of Medicine, Universiti Kebangsaan Malaysia, Kuala Lumpur, Malaysia

## Abstract

**Background:**

Peptic ulcer is a basic term for ulcers on the lower oesophagus, stomach, or jejunum. The specific term for ulcer in the stomach is gastric ulcer. The extensive use of honey around the globe helps researchers to study the usefulness of honey. Many studies had already been conducted and proved the effectiveness of honey in treating gastric ulcer.

**Methods:**

A systematic review of the literature was conducted to identify relevant studies on honey used as an alternative treatment of gastric ulcer cause by NSAIDs. A comprehensive search was conducted in Medline, SCOPUS, and Ebscohost. The main criteria used were articles published in English and using NSAIDs-induced gastric ulcer in rat's model and those reporting the effectiveness of honey.

**Results:**

Articles published between 2001 and 2014 were identified to be relevant in studies related to the inclusion criteria. The literature search found 30 potential and closely related articles in this review, but only 5 articles were taken which meet the criteria needed to be fulfilled.

**Conclusions:**

All studies in this review reported the efficacy of honey for gastric ulcer based on its antioxidant and cytoprotective activities. Most of the studies conducted used different types of honey at various doses on rats. Future studies should be conducted to identify the appropriate dose for humans to achieve similar gastroprotective effects.

## 1. Introduction

Peptic ulcer disease (PUD) is a disease associated with secondary damage caused by secretion of pepsin and stomach acid. The most commonly organs affected by that secretion are the stomach and proximal duodenum. Lower oesophagus, distal duodenum, and jejunum are also affected but less common. About 500,000 patients are reported suffering from PUD every year in the United States alone. It often occurs in patients aged between 25 and 64 years, which is 70% of the cases, costing up to $10 billion a year for treating PUD alone [1]. Since centuries ago, PUD was surgically controlled and caused high morbidity and mortality rates. Until the 1980s, the introduction of histamine H_2_-receptor antagonists (H2RAs) (e.g., cimetidine and ranitidine) led to a decrease in surgery due to PUD by 85% [2].

Specifically, ulcer in stomach refers to gastric ulcer. The most frequent site of ulcer in the stomach is the lesser curvature; however, it can also occur anywhere from pylorus to cardia [3]. Factors such as the environment (cigarettes, alcohol use, and infectious agents), pain killers such as NSAIDs, and life-giving episodes or stress [4] are among the factors that may cause gastric ulcer to patients. In terms of major pathophysiology, prostaglandins (PGs), changes in gastric mucosal barrier and blood flow, and degenerative gastric secretion are contributor to gastric ulcers [5, 6].

Alcohol consumption and cigarette smoking are closely related and become a factor causing chronic inflammation in the stomach. Furthermore,* Helicobacter pylori* (*H. Pylori*) infection is common among alcoholics. Prolonged use of alcohol is also associated with the presence of gastric metaplasia. Disruption of mucous blood flow [7] and angiogenesis and suppression of cell proliferation causes delay in ulcer healing in cigarette smokers [8].

The finding of severe damage to the surface of epithelial cells and sometime until submucosa are found in stress induced gastric ulceration. Antioxidant enzymes in the rat gastric mucosa cause an increase in histamine and pepsin but decrease in gastric fluid with the progress of ulceration (ulcer index) [9].

Under normal circumstances, the stomach is able to withstand highly concentrated hydrochloric acid, reflux bile salts, alcohols, and foodstuffs with varying temperatures and osmolarities. This is due to the ability to repair the damage by the mucosal layer of stomach rapidly if exposed to potentially harmful reasons. The production of PGs is found to modulate most aspects of the mucosal defence, thereby enhancing the resistance of the stomach layer [10, 11]. Endogenous PGs of gastric epithelium and duodenum directly control mucous and bicarbonate secretions, mucosal blood flow, proliferation of epithelial cell, epithelial restitution, and mucosal immunocyte function [12]. Undoubtedly these are the importance of PGs as mucosal defence but in a similar aspect, nitric oxide (NO), can also carry the same function [13]. This means that the suppression of PGs alone does not lead to damage to gastric mucosal [14]. Bioactive PGs production requires enzyme cyclooxygenase (COX). Aspirin and indomethacin are examples of pain killer drugs under nonsteroidal anti-inflammatory drugs (NSAIDs) group; in short, NSAIDs will inhibit the production of the enzyme COX [15], resulting in stunted PGs production [16].

Proton pump inhibitors (PPIs) (i.e., omeprazole, esomeprazole, lansoprazole, and pantoprazole) are widely used to cure gastric ulcer. PPIs work by blocking the adenosine triphosphatase, an important enzyme in the H + and K + exchange process in the final steps of the acid secretory process within the gastric parietal cell. The use of conventional medicine like PPIs is often associated with side effects; for example, peripheral neuropathy is detected as a result of omeprazole therapy [17].

Alternatively, honey is used in treating wounds and stomach-related diseases especially in the Greek, Romans, Chinese, and Egyptian [18]. Honey is known for its sweet taste due to the presence of sugar in its contents. In addition, honey also contains various other substances such as organic acids, proteins, amino acids, vitamins, enzymes, minerals, flavonoids, and antibacterial factors [19].

Despite the efficacy of honey in the cytoprotection of the stomach, honey had also significantly shown effectiveness in protecting against inflammation of the kidneys due to the use of cancer drugs (e.g., Cisplatin) [20], inhibiting or suppressing growth and progression of tumours and cancer without giving any harmfulness or its noncytotoxicity to normal cells [21], used in wound dressing to prevent bacterial growth, thus helping in healing by promoting wound repair [22], and it is found to be efficacious in reducing inflammation in bronchoalveolar lavage fluid and preventing goblet cell hyperplasia in asthma treatment [23].

The presence of high amount of flavonoids in honey is believed to have the value of pharmacological activities including preventing the formation of gastric ulcers via its antisecretory and antioxidant mechanisms [24]. In view of the fact that honey is nutritious and has various medicinal properties, this systematic review was conducted to evaluate the therapeutic potential of honey in the context of its gastroprotective function against NSAIDs-induced gastric ulcers.

## 2. Materials and Methods

### 2.1. Literature Review

Systematic review is conducted to identify past and related studies that use various types of honey NSAIDs-induced ulcer in rat's model from the year 2000 to 2018. Literature search was made by using health sciences journals databases Medline* via* Ovid Medline (articles found were published from year 2000 to 2011), SCOPUS (articles were published from 2001 to 2014), and Ebscohost (articles were published from 2000 to 2011). The search strategy used necessary key words involving a combination of the following set: (1) honey AND (2) (stomach ulcer*∗*＇＇OR ＇＇stomach damage*∗*＇＇ OR ＇＇stomach lesion*∗* AND (3) ＇＇NSAID*∗* OR nonsteroidal anti-inflammatory drug*∗* OR nonsteroidal anti-inflammatory drug*∗*.

### 2.2. Selection of Research Articles

In the search results, some criteria must be met. Only articles (including the abstract) which were published in English language were included. Studies with these characteristics were included: (1) original paper with full text, (2) using NSAIDs as one of ulcer inducers in rat's model, and (3) using honey as treatment. Articles were excluded if they were (1) review articles, (2) written in other language, (3) not using rats (study on profile only), (4) ulcer inducer by other than NSAIDs, (5) duplicated studies, and (6) from news, letter, editorials, or social media.

### 2.3. Data Extraction

Three authors screen the same databases by using the key words in use to ensure that the search results are authentic. In the search process, articles were filtered to ensure that search results fulfilled all the criteria mentioned. Firstly, all the articles that not match the primary studies were removed. The articles in honey study which are not related to gastric ulcer are the highest number removed in this stage. Next, articles that study honey and show significant result lead to the efficacy of treating gastric ulcer, while articles not using rats-induced ulcer model were also removed. Finally, the articles obtained should reflect the method used, as there are articles that use more than one ulcer-inducing method but only presented the selected results which are not NSAIDs-induced ulcer which was also removed from this review.

## 3. Results and Discussion

### 3.1. Selection of Articles

**Figure 1:**
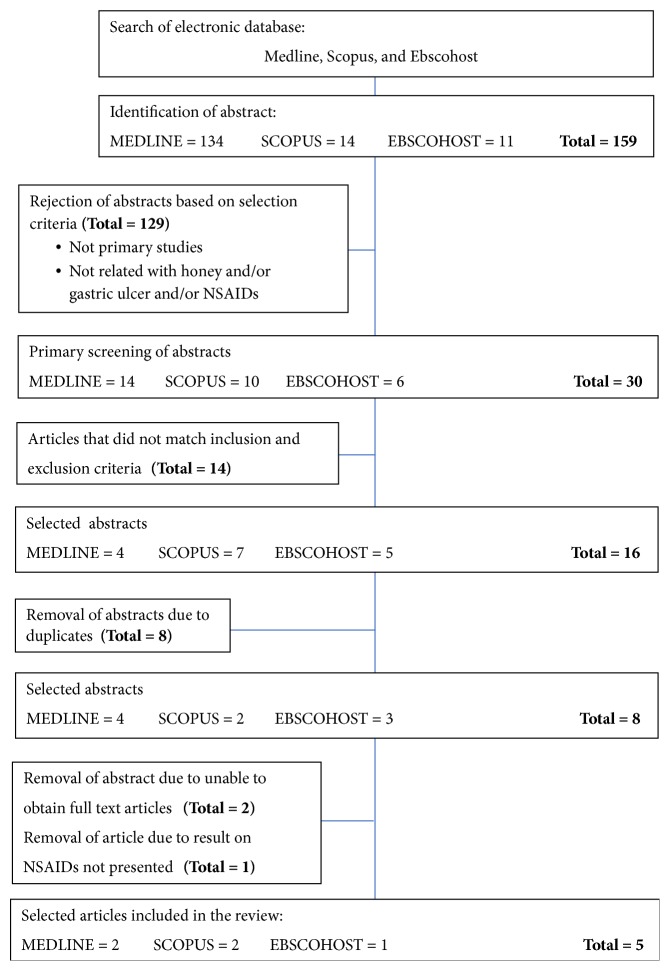
Flowchart to show the selection process of articles in this review.

From the literature searched, 159 articles were identified, 134 were obtained from Medline, 14 were from SCOPUS, and 11 were from Ebscohost. As many as 129 articles are excluded because of not being in primary studies, 14 more articles are rejected after inclusion and exclusion criteria were assessed. Total duplicates articles which are 8 are also removed from this review. The remaining 5 articles fulfil all criteria mentioned and were included in the review. All steps of searching, filtering, and management strategy of selection of articles are shown in Figure 1.

### 3.2. Study Characteristics

All selected studies are published from year 2001 to 2014. In general, all studies using rats induced by NSAIDs have two different focuses; that is, one studied the effects of honey directly on gastric ulcer healing [25], and the other four studies compared the effects of honey with other natural products or substances that are believed to have gastroprotective effect [26–29]. All studies used rats as a model in the experiment. In some papers,* in vitro* study was also presented for some related parameters. Human study is not included in this review. Some of the studies in this review have an interest in studying antioxidants using the same rats' model.

Most studies measured gastric acidity, numbers and diameter of ulcers (by gross and microscopic examination), ulcer index (UI), healing ratio, histological examinations, body weight, antioxidant activities, microvascular permeability (MPV), and myeloperoxidase (MPO) activity,* in vitro *H_2_O_2-_ scavenging activity,* in vitro* superoxide-scavenging activity, acid output, transmural potential difference (PD) study, and osmolality changes. Diversity in how to prepare rat models that are ulcerated using different doses was observed. Duration of study also shows a difference from several hours to several weeks. Rats are induced with NSAIDs agents whether indomethacin or aspirin by using oral gavage, subcutaneous injection (s.c.), or intraperitoneal injection (i.p.). In study [27], induction to ulcer using aspirin is combined with ethanol. Omeprazole [27, 30], sucralfate [26, 31, 32], mannitol [28, 33], and cimetidine [29, 34, 35] have been used in these studies as referral drugs as well as some comparative substances to the efficacy of honey or comparative substances used in the experiments. Different doses used depend on the duration of the experiment and the weight of the rats.

Depending on how many groups are in the study, the number of rats, types, body weight (BW), and sex show the difference from one study to another. In the preparation of the ulcer rat model, Sprague Dawley (SD) [36, 37] and Wistar [25–28] rats were used. A few study reported that growth hormone (GH) has the ability to improve the healing of the gastric ulcer; thus all studies used mature rats that weigh within 150-250 g [38, 39].

Except for chestnut honey, the name of honey used in these studies was not specifically stated; otherwise only wild honey or natural honey is used. The honey doses used were between 1.2 g and 4.25 g per kilogram; body weight (BW) of rat can be observed in this review, while, for study [25], honey doses are calculated in milliosmoles (one thousandth of an osmole) per kilogram (mOsmol/kg), and the doses used are 300 mOsm to 3600 mOsm.

### 3.3. Effects of the Honey in Gastric Ulcer Healing

Nasuti et al. [26] reported, in superoxide-scavenging activity, inhibition of CL absent for all substances except for propolis at 4% concentration in honey-presenting formulation samples. Inhibition activity of Chemiluminescence (CL) measured significantly reduced in the following order: propolis > eucalyptus > ginseng powder > ginseng liquid extractive > royal jelly. The addition of Alimento Supervis (AS) and Alimento Mieleucalipto (AM) reduces the CL value more (64.5% and 65%, respectively) compared to honey alone (38%) obtained from control samples. In the group of indomethacin + honey (1.2 g/kg BW versus 2 g/kg BW), there is no significant difference in terms of UI, MPV, and MPO values.

Adnyana et al. [27] studied the effects of honey, turmeric-honey in combination, and turmeric only to mucosa tissue. In histology reports, reduction of inflammation was observed in all the treated groups. The referral medicines used are omeprazole at 1.8 mg/kg BW. In the aspects of gastric juice parameters, there was no significant difference compared to the omeprazole group as shown in Table 1. With dose of 2125 mg/kg BW, healing of acute ulcer is as good as omeprazole with healing percentage of 49.10% which can be observed. The dose comparison between honey at the higher dose of 4250 mg/kg BW, the healing ulcer effect in terms of diameter, numbers, and index of ulcer is not as good as the low dose used with only 31.44% of healing. CL reduction of AS is 23%, of AM is 19%, and of honey is 10%.

In the review study of Gharzouli et al., [28] in average in absolute ethanol induced group, number of lesions per stomach is 16 with 1 to 12 mm length. Elongation erosions were observed in rat's ulcer induced model. Transmural PD were conducted in 2 difference protocols, detailed as shown in Table 1. In the first experiment protocol, effect of luminal perfusion of isotonic honey (6.36% w/v) on transmural potential difference of rat stomach was observed. During saline perfusion, PD value was recorded at -45.3 ± 0.7 mV and reached -67.5 ± 5.3 mV after 60 minutes of isotonic honey perfusion. At 120 minutes of experiment, 10 mg/kg BW of histamine dihydrochloride was injected through the superior vein of the penis, causing value of PD to drop extensively. Continuous decline of PD value (-29.1 ± 7.3 mV: n = 24) occurred over a period of 15 minutes after acid stimulated by histamine and subsequently a gradual increase occurred but was still below the basal value, while, by using the second study model in this experiment protocol, PD value of perfusion of stomach with isotonic honey showed an increase from -38.5 ± 0.9 mV to -51.4 ± 2.3 mV. The decline was recorded in PD readings after ethanol was added for 5 minutes to sodium chloride (NaCl) perfusion (in control group) reaching -8.7 ± 2.9 mV and isotonic honey perfusion (in treated group) reaching -20.0 ± 2.7 mV. Observation showed that 70% ethanol caused the haemorrhagic lesion of the stomach mucosa, with 543 ± 79 mm2 under saline perfusion and 158 ± 32 mm under the honey treated group, with 70% protection significantly. Acid secretion during saline perfusion remained low and stable at value of 2.52 ± 0.04 *μ*Eq/h and increased acid output was noticed at rate of 6.24 ± 0.29 *μ*Eq/h after the saline was replaced with honey solution. At the point where the histamine was introduced, the increase of acid secretion is significantly observed and this effect persisted for about 1 hour. During the perfusion with honey (30.0 ± 1.1 *μ*Eq/h), acid secretion can be observed to occur more when compared during NaCl perfusion (23.4 ± 1.7 *μ*Eq/h). 57.4% of the total acid is secreted in the honey treated group compared to the control group during 2 hours of data collection.

Endogenous PG-1-2 pg/mg protein (estimated in 6-keto-PG-F-1*α* form) profile obtained from the experiment [27] is used to identify cytoprotection activity in gastric mucosa. From the sample of antrum, natural honey treated group showed 112.68 ± 17.24 (300 mOsm), 170.17 ± 26.99 (600 mOsm), 307.96 ± 47.18 (1800 mOsm), and 395.40 ± 54.62 (3600 mOsm), while in fundus sample, it showed 107.26 ± 18.43 (300 mOsm), 148.62 ± 18.43 (600 mOsm), 234.14 ± 33.27 (1800 mOsm), and 367.54 ± 50.72 (3600 mOsm). Result from sample of fundus is shown to be slightly lower in all doses of honey compared to antrum sample. In the mannitol treated group, sample of antrum showed 70.03 ± 9.81 (300 mOsm), 93.53 ± 13.02 (600 mOsm), 356.45 ± 52.48 (1800 mOsm), and 415.90 ± 60.16 (3600 mOsm), while in fundus sample, it showed 59.48 ± 8.04 (300 mOsm), 53.85 ± 7.79 (600 mOsm), 217.25 ± 31.39 (1800 mOsm), and 400.98 ± 58.44 (3600 mOsm). The result observed showed a drastic elevation of value from 600 mOsm to 1800 mOsm in mannitol group compared to honey. In the absolute control group it can be observed that 281.10 ± 39.16 in antrum and 165.11 ± 23.41 in fundus are lower than those in both honey and mannitol (1800 mOsm and 3600 mOsm). In indomethacin control group, it is significantly lower in all treated groups and absolute control group with 47.01 ± 9.05 from antrum sample and 55.02 ±11.32 from fundus sample.

Bukhari et al. [29] reported that, in animals treated with* nigella sativa* (NS), healing activity against gastric ulcer is similar to the group treated with natural honey. The gastric ulcer from honey treated group healed in 14 rats (78%) in this experiment. The same 14 rats also were observed to have regeneration of inflamed gastric mucosa compared to 13 rats from the NS treated group and 16 from cimetidine treated group. In total, 4 rats retained are gastric lesions state from honey treated group with recovery of 78% when examined microscopically.

### 3.4. Effects of Honey with Combination Agents in Gastric Ulcer Healing

Nasuti et al. [26] reported that AS, AM, and honey have an extraordinary antioxidant activity. In pretreated animals with doses of 2 g/kg of honey, AS, and AM, the results are significant to reduce UI and MPV. Comparisons made with low doses (1.2 g/kg) of honey, AS, and AM did not show the same protective effect as sucralfate but macroscopically, gastric lesions were decreased and sufficient reduction of neutrophil infiltration was shown. In the combination of honey (2125 mg/kg BW) and turmeric (135 mg/kg BW) group, ulcer healing by 31.51% was observed as compared to 20.78% healing from turmeric alone (Adnyana et al. [27]).

## 4. Discussion

This review reveals the benefits of honey in treating gastric ulcer induced by NSAID. Result obtained from Adnyana et al. [27] concluded that low dose of honey (2125 mg/kg) is better in gastric ulcer healing than the higher dose of 4250 mg/kg as mentioned in Table 1. This could be due to the nature of properties of honey itself with pH of 3.88 which could contribute to the increase acidity of gastric juice which could irritate the gastric mucosa of the stomach (Supijona et al. [40]). The gastric acidity shows no significant difference compared to the referral drug used; ulcer healing of honey is different from omeprazole of which mechanism of action is via its antisecretory properties. Since antisecretory drugs are reported to have side effects [17, 41] after long terms used, honey in suitable doses is suggested as a potential alternatively. In another study, turmeric shows good effects on ulcer healing if combined with honey rather than turmeric alone; thereby confirming plant extracts combination with honey can increase outcome therapy of gastric ulcer [42]. The presence of antioxidants activities detected in some previous studies [43–45] played an important role in the treatment of gastric ulcer by curcumin, a chemical found in the turmeric [46]. It is the same effect of matrix metalloproteinases (MMPs) of curcumin that plays a role in extracellular matrix degradation and remodelling during inflammation and wound healing process. The BW of the rats given turmeric or combination of turmeric with honey increases, thus suggesting that the appetite is not altered even after induction of gastric ulcer as compared to rats in group without treatment. This could be due to the reduced damage in the gastric microenvironment with honey treatment, thus reducing the loss of BW otherwise observed in nontreated rats which develops severe gastric ulceration.

Reduced in neutrophil infiltration into gastric mucosa is also parallel to gastric protection, as reported by Nishida* et al*. [47]. It can be observed with higher dose (2 g/kg) group of (indomethacin + AS) and (indomethacin + AM) probability by their anti-inflammatory ability [26]. Inhibition of xanthine oxidase by 2 phenolic compounds of propolis, galangin, and caffeic acid phenethyl ester [48] appears to reduce neutrophil infiltration in the gastric mucosa by hindering the activation pathway of neutrophils [49]. Free radical scavenging profile and modulation of leukocyte function show gastroprotection activity against indomethacin treatment. MPO activity is increased by indomethacin treatment [44] which was reduced by propolis [26]. The results obtained from the* in vivo* study showed that the gastroprotective efficiency of honey was also increased by adding propolis in the formulation. Treated groups with low dose (1.2 g/kg) of honey, AS, and AM also showed a similar effect on MPO activity, suggesting only the anti-inflammatory effects of honey as the contributing factor, while, at high dose (2 g/kg), the activity of MPO in group of indomethacin + AS and indomethacin + AM was significantly decreased probably because of the higher dose of propolis used compared in low dose (1.2 g/kg) group. Significant results also can be obtained from* in vivo *and in* vitro* study at a higher dose (2 g/kg) of indomethacin + AS and indomethacin + AM group in gastroprotective action. However, at the lower dose (1.2 g/kg) group, it is not showing the same pattern. For that, addition of natural products to honey as supplements does not enhance the gastroprotection action of honey, because honey alone can provide the full protection.

In the study by Gharzouli et al. [28], gastric ulcers were induced by either ethanol, indomethacin, or ASA-HCL as necrotizing agents. By using monofloral honey, polyfloral honey, and glucose-fructose-sucrose-maltose (GFSM) mixture (prepared by mixing all with same proportions as honey: 17.5 ml of distilled water, 38.2 g of fructose, 31.3 g of glucose, 1.5 g of sucrose, and 7.3 g of maltose) as gastroprotective agents, the three agents were compared for effectiveness in lowering lesion formation in various rat models. Base on the result, Gharzouli* et al*. [28] claimed that gastric instillation of honey or honey like solution (i.e., GFSM) shows positive outcomes with all results obtained suggesting that oral administration of GFSM mixture or honey has the ability to prevent lesions of gastric mucosa induced by either ethanol, indomethacin, or ASA-HCL. Comparison of protection of the gastric mucosa from ulcerogenic agent shows that about 90% protection is obtained from models using ethanol and acidified ASA while the protection was only as much as 64% in indomethacin model. The results comply with the report of [28, 33, 50, 51]. Transmural PD in a stable state and luminal surface of mucosa is electronegative to serosa surface, respectively, if the gastric barrier is intact [52]. Any damage at that barrier by any damaging agents will cause a decrease of PD and mucosal surface is also altered [53]. Cytoprotective activity can be seen in perfusion of stomach with a mixture of honey-ethanol with gastric lesions reduction up to 70%. Honey induces acid and histamine stimulation in the stomach. Acid that results from histamine stimulation will decline the PD value. This result is in agreement with data of substances which are able to modify gastric acid secretion and variation in gastric PD in animal and in man [53].

Bukhari et al. [29] found that, after 2-week treatment with honey, 33% of animals were having mild acute and chronic inflammation with neutrophils, lymphocytes, and macrophages infiltrated while the remaining 66% of animals showed gastric ulcer recovery activity. In 6 weeks of honey treatment, 1 animal (16.6%) showed smooth mucosa surface on gross examination, and microscopically also one animal showed sign of chronic inflammation with fibrosis, while 5 remaining animals had no lesions. In total, 77.7% (14/18 animals) showed complete remission from ulceration, inflammation, and erosions produced from NSAIDs, which showed natural honey, are effective in gastric ulcer healing.

## 5. Strength and Limitation of This Review

Many researchers had identified the effectiveness of honey in many areas with promising result, thus putting honey as a great natural substance with tons of benefits. Alternative and natural substances are widely used in many diseases offering lesser side effects compared to known drugs used in such particular conditions. As gastric ulcer is a global issue, especially with the increased use of NSAIDs for pain management, seeking an alternative method to reduce gastric side effects is warranted. This review is highly relevant to identify the effectiveness of honey on gastric ulcer treatment. As shown in this review, the effects of honey on gastric ulcer induced in rats by NSAIDs are positively shown. For the benefit of comparison and to aim clearer understanding, only articles on rats-induced model were chosen to compare the ulcer index and cytoprotective activities. Nevertheless, one paper which presented* in vitro* study parameter is not excluded from the review because an in* vivo* parameter on same rats was also studied [26].

One of the limitations of the research on honey is the fact that they come in various colours, viscosities, tastes, and different concentration of potential active substances. The honey used in the articles review did not clearly specify the name of the honey or whether it is from natural source or wild. The difference of honey doses may also contribute to different findings observed, for instance, 1.2 g/kg of honey [26] versus 4250 mg/kg [27]. In some parameters, the comparison on the outcomes seems difficult because it is not presented in other articles. The duration of treatment can also contribute to the limitation as maturation of rats can contribute to ulcer healing by GH [38, 39]. Rats fasting duration before being sacrificed in articles reviewed are also different from one study to another, with one paper [29] which did not specify the fasting period.

## 6. Recommendation

The standardization of honey in concentration plays the important bench mark as researcher can compare the effectiveness of various honey at the same dose. The origin of the honey is important to identify the different honey used in a particular research. Treated and pretreated rats model with various duration of treatment need to be well designed to provide better outcome and understanding of the study.

## 7. Conclusion

Honey as a natural agent has a gastroprotective potential as shown by the outcomes from various studies. It provides mucosa healing mainly via its antioxidants, anti-inflammatory, and cellular protective mechanism. However, further studies are required to determine the potential active substances in honey that contributes to gastric healing and the potential use of honey in human.

## Figures and Tables

**Table 1:** Characteristics of Studies in this Review.

**Study 1**	**Ulcer-Induced / Dose**	**Rats / Fasted**	**Group / Treatment Dose and Duration**	**Methodology**	**Result**

Adnyana *et al.* (2014)	**Aspirin** 405 mg in 1% Carboxymethyl cellulose sodium (CMC-Na) **Ethanol** 95% (1 ml/200 g)	35 Male albino Wistar 150 – 250 g 1 night before sacrificed	7 Groups of 5 animals each. (1) Control group – water given (2) Induced ulcer for 10 days without treatment (3) Treatment group – honey 2125 mg/kg (4) Treatment group – honey 4250 mg/kg (5) Treatment group – Turmeric 135 mg/kg (6) Treatment group – honey 2125 mg/kg + Turmeric 135 mg/kg (7) Treatment group – Omeprazole 1.8 mg/kg Total 10 days experiment	405 mg in 1% CMC-Na, from day 1 – 10, and on day 5 and 95% ethanol (1 ml/200 g). Treatment given from day 6 until day 10. Turmeric Rhizome grinded and boiled at 90°C for 15 min. Honey is diluted with distilled water.	Erosion and inflammation of mucosa lesser in test group than in control group. Decline in body weight (BW) of rats in control group but not in honey and/or with turmeric group. Healing ability in group 3, 4, 6 and 7 are consistent with histological result. No significant difference in gastric acidity parameter except for group 7. Diameter, number and index ulcer shown that treatment with lower dose honey is more significant in ulcer healing compare to treatment with higher dose honey. 31.51% ulcer healing obtained in group 6 (effect is lower than group 3 but higher than group 5).

**Study 2**	**Ulcer-Induced / Dose**	**Rats / Fasted**	**Group / Treatment Dose and Duration**	**Methodology**	**Result**

Nasuti* et al. *(2006)	**Indomethacin** 60 mg/kg.	Male Wistar 150 – 170 g 24 hours before sacrificed	9 groups of 18 animals each. (1) Control group – water given (2) Indomethacin (ulcerated group) (3) Indomethacin + 1.2 g honey (4) Indomethacin + 2 g honey (5) Indomethacin + 1.2 g AS (6) Indomethacin + 2 g AS (7) Indomethacin + 1.2 g AM (8) Indomethacin + 2 g AM (9) Indomethacin + 100 mg Sucralfate Total 7 days experiment.	Indomethacin 60 mg/kg is induced 30 minutes after daily treatment orally. Alimento Supervis (AS) containing royal jelly 2%, ginseng powder 4%, ginseng liquid extractive 4% and hydroalcoholic propolis extract 4%. Alimento Mieleucalipto (AM) containing hydroalcoholic propolis 4% and eucalyptus 4%. Both AS and AM derived from same chestnut honey. Evaluation of gastric lesions by UI. Honey, AS, and AM solubilized in distilled water (4% solution). Ginseng, propolis, eucalyptus, and royal jelly dissolved in hydroalcoholic solution (4%, 16%, and 32% respectively).	UI value (1) In indomethacin group is 35.6 ± 1.04 vs 0.0 ± 0.00 in control group. (2) Sucralfate, 2 g/kg of honey, AS, and AM decrease UI to (3.5 ± 1.04, 4.33 ± 2.27, 4.25 ± 0.95 and 3.25 ± 0.25 respectively) (3) With low honey dose of 1.2 g/kg of honey, AS and AM represent of (23.55 ± 3.63, 18.46 ± 3.39 and 15.08 ± 2.72 respectively) MPV value (1) 5.09 ± 0.37 *μ*g EB/g tissue is obtained in indomethacin group vs 2.08 ± 0.47 *μ*g EB/g tissue from control group. (2) In sucralfate group, 3.27 ± 0.55 *μ*g EB/g tissue. (3) 2 g/kg of honey, AS, and AM shown (3.44 ± 0.61, 3.04 ± 0.78, 3.15 ± 0.31 *μ*g EB/g tissue respectively) (4) No reduction of value in 1.2 g/kg of honey, AS, and AM observed compared with indomethacin group. MPO value (1) Increase MPO activity in indomethacin compared to control (11.82 ± 0.31 vs 7.54 ± 0.61 U/g tissue respectively) (2) 2 g/kg of honey, AS, and AM shown (9.21 ± 0.67, 1.31 ± 0.19, 2.07 ± 0.20 U/g tissue respectively) (3) 1.2 g/kg of honey, AS, and AM shown (5.8 ± 0.24, 8.03 ± 0.25, 6.02 ± 0.87 U/g tissue respectively)

**Study 3**	**Ulcer-Induced / Dose**	**Rats / Fasted**	**Group / Treatment Dose and Duration**	**Methodology**	**Result**

Gharzouli *et al.* (2002)	**Indomethacin** 30 mg in 0.25% carboxymethyl cellulose (CMC) + 2 drops of Tween 80 per 10 ml **Aspirin -HCL (ASA-HCL)** 300 mg dissolved in 0.15 N HCL containing 0.25% w/v **100% ethanol** 5 ml/kg	Either sex Wistar 200 – 250 g 48 hours before sacrificed	8-10 animals per groups Indomethacin induced ulcer (1) Indomethacin + 2.5 g/kg of monofloral honey (2) Indomethacin + 2.5 g/kg of polyfloral honey (3) Indomethacin + GFSM mixture 50% w/v (4) Control group receive 5 ml water /kg Ethanol induced ulcer (5) Ethanol + 2.5 g/kg of monofloral honey (6) Ethanol + 2.5 g/kg of polyfloral honey (7) Ethanol + GFSM mixture 50% w/v (8) Control group receive 5 ml water /kg ASA-HCL induced ulcer (9) ASA-HCL + 2.5 g/kg of monofloral honey (10) ASA-HCL + 2.5 g/kg of polyfloral honey (11) ASA-HCL+ GFSM mixture 50% w/v (12) Control group receive 5 ml water /kg	Honey or glucose – fructose – sucrose – maltose (GFSM) mixture is given just before injection of indomethacin. Aspirin, by oral gavage 30 minutes after pretreatment with test solution. Killed after 3 hours. 100% ethanol (5ml/kg) by oral gavage 30 minutes after pretreatment with test solution. Killed after 15 min. Transmural potential difference (PD) obtained between gastric lumen and peritoneal cavity with potentiometric recorder. Acid output evaluated by titration to pH 7 using 0.01 N NaOH. Model study 1 (1) 30 min require for PD stabilization. 210 min perfusion of saline in control group. (2) In treated group, 60 min infuse with saline, then 60 min with isotonic honey, then again with saline for 90 min. (3) At 120 min, histamine dihydrochloride (10 mg/kg) is injected by bolus via superior vein of the penis. Model study 2 (1) 30 min require for PD stabilization. (2) Then perfuse with isotonic honey for 60 min. (3) During period of 15 min, absolute ethanol is added for 5 min to give final 70% concentration. Then, replace the solution with the same preceding preparation. (4) Control group receive saline with the same protocol of honey.	Total lesions length (mm) (1) In control group, 52.7 ± 6.9, 11.4 ± 2.2, and 37.8 ± 9.7 observed from ethanol, indomethacin and ASA-HCL respectively. (2) Indomethacin-induce group (6.7 ± 1.2 from GFSM mixture group, 4.1 ± 0.6 from monoflora honey group and 5.1 ± 0.6 from polyflora honey group) are measured. (3) ASA_HCL induce group, 4.6 ± 1.5, 3.1 ± 0.6 and 2.8 ± 1.7 are obtained from GFSM mixture, monoflora honey and polyflora honey respectively. (4) In ethanol induce group, GFSM mixture group shown 1.0 ± 0.4, monoflora honey 0.8 ± 0.5, and polyflora honey 1.0 ± 0.5.

**Study 4**	**Ulcer-Induced / Dose**	**Rats / Fasted**	**Group / Treatment Dose and Duration**	**Methodology**	**Result**

Gharzouli *et al.* (2001)	Indomethacin 30 mg/kg	50 female Wistar of 210 – 220 g 24 hours before sacrificed	5 animals per group. (1) Absolute (abs) control – normal saline given (2) Indomethacin control (3) 300 mOsm honey (4) 600 mOsm honey (5) 1800 mOsm honey (6) 3600 mOsm honey (7) 300 mOsm mannitol (8) 600 mOsm mannitol (9) 1800 mOsm mannitol (10) 3600 mOsm mannitol	Indomethacin dose is 30 mg/kg by i.p suspended in normal saline with 30 mg/ml given to animals at the beginning (0 min) In treated with honey or mannitol groups, doses are given at 0. Min and 2 hours. At 4th hour, animal sacrificed by cervical dislocation and stomach is taken for UI scoring. Endogenous prostaglandin 1-2 (PG-1-2) determined by radioimmunoassay (RIA) of oxyntic cell (antrum and fundus of stomach)	UI value (1) Absent in abs control group (2) Indomethacin group shows 13.3 ± 4.2 (3) In honey treated group, (5.5 ± 2.9, 1.2 ± 0.5, 1.6 ± 0.7, 1.0 ± 0.5 are observed from dose of 300 mOsm, 600 mOsm, 1800 mOsm and 3600 mOsm respectively. (4) In mannitol treated group, (5.3 ± 2.4, 1.5 ± 0.7, 1.5 ± 0.6, 1.0 ± 0.4 are observed from dose of 300 mOsm, 600 mOsm, 1800 mOsm and 3600 mOsm respectively.

**Study 5**	**Ulcer-Induced / Dose**	**Rats / Fasted**	**Group / Treatment Dose and Duration**	**Methodology**	**Result**

Bukhari et al. (2011)	Aspirin 200mg	100 male albino rats of 200 - 250 g	5 groups with 20 animals each. Group A: Control group 2ml of 2% gum tragacanth (GT) Group B: Nigella sativa group (30 mg/kg) Group C: Natural honey group (30 mg/kg). Group D: Cimetidine group (0.2 g/kg) Group E: Acetylsalicylic group (0.2 g/kg)	20 rats from 2ml of 2% GT 2 rats from all groups (group A given 2ml of 2% GT, group B, C, D, and E given 0.2 g/kg of acetylsalicylic) and sacrificed after 3 days for gastric lesions confirmation. The remaining rats divided into subgroups (1, II, and III and sacrificed after 2 weeks, 4 weeks, and 6 weeks respectively) and give the treatment accordingly. All chemical used suspended in 2% GT aqueous solution. Honey diluted in distilled water and administered orally with 0.5 ml/100 g BW.	By the gross examination, number of animals with lesion from each group: (1) AI – AIII = 0 (2) BI = 1, BII = 1, and BIII = 1 (3) CI = 1, CII = 1, and CIII = 1 (4) DI = 0, DII = 1, and DIII = 0 (5) EI = 5, EII = 6, and EIII = 6 By the gross examination, number of animals with lesion from each group: (1) AI – AIII = 0 (2) BI = 2, BII = 1 and BIII = 2 (3) CI = 2, CII = 2 and CIII = 1 (4) DI = 1, DII = 1, and DIII = 0 (5) EI = 5, EII = 6 and EIII = 6
